# Supplementary material for: Trends in Simple and Complex Appendicitis in Children and the Potential Correlation to Common Viral Pathogens—A Retrospective Cohort Study between 2010 and 2019 in The Netherlands
Source: Children (Basel). 2023 Dec 11;10(12):1912. doi: 10.3390/children10121912 (PMC10741496; doi:10.3390/children10121912)
Supplement: Supplementary file 1 [file children-10-01912-s001.zip › Table S1.pdf]

Table S1. Articles addressing seasonal variation in children with appendicitis.

Author (year)	Country	Number patients	Age (years)	Season	Finding
**Deng (2010)** [17]	USA	31.457	< 18	Spring (Mar-May)	24.2%
				Summer (Jun-Aug)	25.5%
				Fall (Sep-Nov)	25.8%
				Winter (Dec-Feb)	24.6%
**Zhang (2018)** [16]	China	3.436	< 18	Spring	
				Summer	Peak
				Autumn	Peak
				Winter	
**Rautava (2018)** [15]	Finland	8494	< 16	Spring (Mar-May)	24.9%
				Summer (Jun-Aug)	27.2%
				Autumn (Sep-Nov)	24.1%
				Winter (Dec-Feb)	23.8%
**Jangra (2013)** [18]	India	395	-	Rainy season (Jul-Sep)	Peaks
**Hsu (2019)** [19]	Taiwan	171	< 18	Summer (May-Oct)	59.1%
				Non-Summer (Nov-Apr)	40.9%
**Gallerani (2006)** [20]	Italy	654	≤ 19	Spring	23.5%
				Summer	21.7%
				Autumn	25.5%
				Winter	29.2%
